# Artificial Intelligence–Based Radiotherapy Contouring and Planning to Improve Global Access to Cancer Care

**DOI:** 10.1200/GO.23.00376

**Published:** 2024-03-14

**Authors:** Laurence E. Court, Ajay Aggarwal, Anuja Jhingran, Komeela Naidoo, Tucker Netherton, Adenike Olanrewaju, Christine Peterson, Jeannette Parkes, Hannah Simonds, Christoph Trauernicht, Lifei Zhang, Beth M. Beadle

**Affiliations:** ^1^ University of Texas MD Anderson Cancer Center, Houston, TX; ^2^ Guy's and St Thomas Hospitals, London, United Kingdom; ^3^ Stellenbosch University, Stellenbosch, South Africa; ^4^ University of Cape Town, Cape Town, South Africa; ^5^ Stanford University, Stanford, CA

## Abstract

**PURPOSE:**

Increased automation has been identified as one approach to improving global cancer care. The Radiation Planning Assistant (RPA) is a web-based tool offering automated radiotherapy (RT) contouring and planning to low-resource clinics. In this study, the RPA workflow and clinical acceptability were assessed by physicians around the world.

**METHODS:**

The RPA output for 75 cases was reviewed by at least three physicians; 31 radiation oncologists at 16 institutions in six countries on five continents reviewed RPA contours and plans for clinical acceptability using a 5-point Likert scale.

**RESULTS:**

For cervical cancer, RPA plans using bony landmarks were scored as usable as-is in 81% (with minor edits 93%); using soft tissue contours, plans were scored as usable as-is in 79% (with minor edits 96%). For postmastectomy breast cancer, RPA plans were scored as usable as-is in 44% (with minor edits 91%). For whole-brain treatment, RPA plans were scored as usable as-is in 67% (with minor edits 99%). For head/neck cancer, the normal tissue autocontours were acceptable as-is in 89% (with minor edits 97%). The clinical target volumes (CTVs) were acceptable as-is in 40% (with minor edits 93%). The volumetric-modulated arc therapy (VMAT) plans were acceptable as-is in 87% (with minor edits 96%). For cervical cancer, the normal tissue autocontours were acceptable as-is in 92% (with minor edits 99%). The CTVs for cervical cancer were scored as acceptable as-is in 83% (with minor edits 92%). The VMAT plans for cervical cancer were acceptable as-is in 99% (with minor edits 100%).

**CONCLUSION:**

The RPA, a web-based tool designed to improve access to high-quality RT in low-resource settings, has high rates of clinical acceptability by practicing clinicians around the world. It has significant potential for successful implementation in low-resource clinics.

## Teaser

The RPA generates acceptable radiotherapy contours and plans, as judged by 31 doctors in six countries on five continents.

## INTRODUCTION

Cancer cases worldwide are expected to soar to over 24 million by 2030, with the largest growth in low- and middle-income countries (LMICs).^1^ Radiotherapy (RT) is a cost-effective cancer treatment; over 50% of patients in high-income countries receive RT during their treatment course.^2^ However, over 90% of the population in low-income countries lack access to RT.^3^ The ability to address global cancer care cannot avoid discussions of RT access and quality.

**CONTEXT**

**Key Objective**
To determine if radiotherapy (RT) autocontours and autoplans created by the Radiation Planning Assistant, a fully automated suite of RT contouring and planning tools, are clinically acceptable by physicians around the world.
**Knowledge Generated**
Contours and plans were created for cancers of the head/neck, cervix, breast, and whole brain. Extensive physician review, mimicking a real-life workflow, demonstrates that these tools are clinically acceptable for patient care, with no or minor edits.
**Relevance**
The growing burden of cancer is disproportionately affecting patients in low- and middle-income countries where access to RT is limited by hardware and staffing issues. This study demonstrates that automation can produce clinically acceptable RT contours and plans for a diverse group of clinics, patients, and practice settings throughout the world.

Extensive analyses of the RT needs of LMICs have often focused on the necessary investment in hardware. The International Atomic Energy Agency (IAEA) calculated that only 52% of nations in Africa had access to external-beam RT, 39% to brachytherapy, and no country had the capacity to meet its population need.^4^ The Lancet Oncology Commission estimated that it would require $184 billion US dollars (USD) to scale up RT to meet needs in LMICs from 2015 to 2035, with a projected net benefit of $278.1 billion USD over the same period.^5^ Hence, the capital investment in RT, while substantial, is anticipated to be cost-effective. Furthermore, investment in cancer treatment, imaging, and quality of care is estimated to avert 7% of cancer deaths worldwide.^6^

In addition to hardware availability, the lack of human resources, namely trained radiation therapists, medical physicists, and radiation oncologists, portends continued issues with RT availability, even in those centers with equipment. In 2014, it was estimated that there would be a global dearth of over 29,000 radiation therapists, 9,000 medical physicists, and 12,000 radiation oncologists.^7^ Further studies have shown the considerable needs for education,^8-10^ implementation,^11^ and resource-specific treatment guidelines.^12^

One initiative to improve global RT access is automation, by potentially reducing the number of highly skilled staff that must be trained and making those on hand more efficient. These methods promise potential time-savings for target delineation and treatment planning.^13,14^ Automation, namely deep learning and artificial intelligence (AI) approaches, have been increasingly common in medicine over the past decade,^15^ although these have focused on workflows, staffing, and funding in high-income regions.

The Radiation Planning Assistant (RPA) is a fully automated RT contouring and planning system that can provide high-quality RT solutions for low-resource centers around the world.^16,17^ The RPA is designed to be agnostic to equipment and software at each site; as a remotely accessed website, it can be used for contouring and planning regardless of the local vendor. As of May 2023, the RPA has been US Food and Drug Administration (FDA) 510(k) cleared, not yet being marketed. It has been developed in partnership with LMIC users, with the goal of creating a tool that will help scale their efforts to treat more patients with high-quality RT.

The original development of the RPA focused on cancers of the cervix, breast, and head/neck; whole-brain RT was also added. Although analysis of the targets, normal tissues, and fields/plans can be done geometrically using predetermined metrics, each physician fundamentally needs to determine if they will choose to use these contours and plans for their patient. Previous work has shown variability of these approaches from different physicians,^18-20^ even with guidelines.^21^ Metrics may not predict whether the resultant AI-generated contours and plans/fields are clinically acceptable; physicians need to judge what should be delivered to their patients.

In this study, we have assessed the clinical acceptability of RPA-generated contours, fields, and RT plans through a comprehensive review by physicians around the world. These analyses focused on cases of head/neck cancer, cervix cancer, and breast cancer, as well as whole-brain RT, as these encompass multiple RT techniques and levels of complexity.

## METHODS

### Automated Workflows

**FIG 1:**
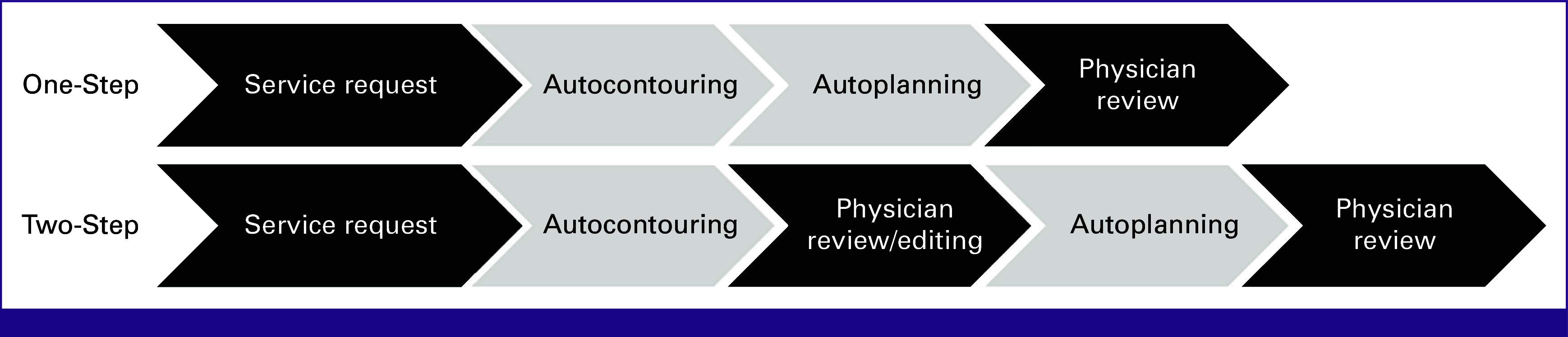
Schematic of one-step and two-step workflows of Radiation Planning Assistant. The physician involvement is shown in the dark fields.

The RPA system uses one-step and two-step workflows, depending on the level of complexity of the cases (Fig 1). The one-step workflow is an end-to-end automated process, in which the contours and the plan are generated in a single step. The treating physician makes specific selections at the start of the process (eg, targets and dose) and at the end (eg, edits and final plan review). The two-step workflow represents a more typical workflow, where contours are generated and approved, and then the RT plan is generated and approved. This level of automation is appropriate for highly conformal planning, where automatically generated targets may require editing before planning.

Users access these services from the RPA website,^22^ requiring upload of computed tomography images and download of the RPA outputs (contours and plans). This low-cost approach was selected to make the tool widely available without the need for local installation or upgrades. To ensure that the plans are correct for the local treatment device, the user must recalculate the RPA plans in their own treatment planning system; this also ensures that the plans enter the local clinical workflow, with clinical review and approval. Extensive risk assessment has demonstrated the vital role of the final reviews in this process.^23,24^

### Algorithms and Models

The RPA uses AI algorithms to generate contours and treatment plans, with dose calculations and other functions provided by a commercial treatment planning system (Eclipse, Varian Medical Systems); however, the individual LMIC clinic does not need to have this software program. Specific tasks necessary for creation of contours and plans are as follows.

#### Isocenter Placement

The marked isocenter is automatically localized from the positions of 3-point external fiducial markers, and the body is automatically contoured.

#### Cervical Cancer: Four-Field Box Plans on the Basis of Bony Pelvic Anatomy (one-step workflow)

These are relatively simple plans recommended by ASCO and the IAEA for the treatment of cervical cancer in low-resource settings.^12,25^ The RPA creates these plans in a single step. First, the pelvic bones are contoured using deep learning models. This anatomy is then projected into each beam's eye view, and the shape of the treatment field apertures are designed on the basis of visible bony landmarks. Dose is then calculated, and beam weights optimized to give a homogeneous dose distribution.

#### Cervical Cancer: Four-Field Box Plans on the Basis of Soft Tissue (one-step workflow)

These are also simple plans that are straightforward to review and treat. The advantage of this approach is that it does not rely on assumptions about the geometric relationships between the targets and bony landmarks; the disadvantage is that they require more physician time. The RPA automatically generates the target contours, generates treatment field shapes, and optimizes the beam weights in a single step.

#### Cervical Cancer: Volumetric-Modulated Arc Therapy Plans (two-step workflow)

Volumetric-modulated arc therapy (VMAT) planning changes the treatment field shapes while rotating the gantry. The advantage of this technique is that it allows conformal dose distribution to the targets and reduced dose to normal tissues. However, this approach requires advanced treatment devices and significant human and equipment resources for plan preparation and quality assurance. The RPA automatically generates the target contours. The treating physician must review, edit, and approve the contours before the automatic generation of a treatment plan.

#### Breast Cancer—Postmastectomy: Multiple-Field Three-Dimensional Conformal Plans (one-step workflow)

RT planning for postmastectomy breast cancers requires a complex combination of matched radiation fields and techniques to improve dose homogeneity. The RPA creates a plan in a single step. First, targets and normal tissues are automatically contoured. Then, support vector machine classification determines the entry angles for the treatment fields; this allows treatment of the chest wall and supraclavicular lymph nodes while minimizing dose to the lung, heart, trachea, and spinal cord. Next, treatment field shapes are calculated from beam's-eye-view projections of the healthy tissues. Finally, small subfields are added to the plan to improve dose homogeneity (known as field-in-field).

#### Head/Neck Cancer: VMAT Plans (two-step workflow)

VMAT for the treatment of head/neck cancer requires multiple target dose levels and challenges because of the number of adjacent critical normal tissues. The RPA generates these plans in a two-step process. The treating physician indicates the desired elective nodal coverage (elective clinical target volumes [CTVs]), and autocontours for the elective CTVs and normal tissues are then generated. The treating physician then delineates the gross tumor volume (GTV) and highest dose CTV (CTV1), and reviews and edits (as needed) the autocontoured CTVs and the autocontoured normal tissues. Once the contours are approved, VMAT plans are automatically generated and available for review and approval.

#### Whole Brain: Opposed Lateral Plans on the Basis of Bony Anatomy (one-step workflow)

Whole-brain treatments typically involve opposed lateral beams, with fields designed to reduce dose to some normal structures, such as the lenses of the eye. The RPA creates plan in a one-step workflow, similar to cervical cancer. There are two approaches that are commonly used to avoid the normal structures: (1) using the multileaf collimators or (2) rotating the collimator (main field/jaws). We developed automatic solutions for both approaches.

#### Verification Algorithms

Quality assurance review of contours and treatment plans is extremely important to ensure that errors do not result in incorrect patient treatments. Automation of these quality assurance tasks can support the human plan review process and reduce risk. Specifically, the RPA includes primary models used to generate the actual treatment plan, and verification models that are used to check the treatment plan, on the basis of the concept that two independent approaches are unlikely to fail in the same way. These include determination of isocenter, contours, and field apertures. They are either trained with different AI architectures or use completely different automated algorithms. Approach 1 and approach 2 (Tables 1 and 2) represent the two different algorithms designed to be quality checks on each other. Of note, there is only a single approach used for target structures (Table 1) since variation in these likely reflects treatment philosophy differences rather than a true error.

**TABLE 1:** Acceptability of Radiation Planning Assistant Autocontouring of Normal Tissues and Targets

Organ	Normal Tissues				Targets		
	Approach 1^a^		Approach 2^a^		Target Region	% Use as-is	% Use as-is or After Minor Edits
	% Use as-is	% Use as-is or After Minor Edits	% Use as-is	% Use as-is or After Minor Edits			
Brain	99	100	100	100	Retropharyngeal lymph nodes	83	96
Brainstem	97	100	80	96	Lymph nodes II-IV	56	90
Cochlea	98	100	99	100	Lymph nodes IA-V	47	92
Esophagus	93	97	93	99	Lymph nodes IB-V	49	93
Eye	100	100	99	100	Lymph nodes II-V	59	92
Larynx	71	96	91	97			
Lens	88	89	89	89			
Mandible	100	100	100	100			
Optic chiasm	49	87	95	100			
Optic nerve	88	97	98	100			
Parotid gland	74	88	100	100			
Spinal cord	100	100	99	100			
Submandibular gland	76	80	87	92			
Bladder	85	93	75	89	Primary CTV	33	75
Femoral head	99	100	100	100	Nodal CTV	83	92
Kidney	95	98	97	99	PAN	88	93
Liver	77	88	—	—			
Rectum	85	97	40	91			
Spinal cord	100	100	96	100			
Lung	91	99	—	—			
Heart	56	91	—	—			

Abbreviations: CTV, clinical target volume; PAN, para-aortic lymph nodes.

^a^ Approaches 1 and 2 represent different algorithms that serve as cross-checks on each other.

**TABLE 2:** Acceptability of Radiation Planning Assistant Automated Treatment Plans

Plan Type	Approach 1^a^		Approach 2^a^	
	% Use as-is	% Use as-is or After Minor Edits	% Use as-is	% Use as-is or After Minor Edits
Head and neck VMAT	87	96	—	—
Cervix four-field box (bones)—field shape	93	97	91	100
Cervix four-field box (bones)—dose distribution	81	93	—	—
Cervix four-field box (soft tissue)—field shape	86	98	—	—
Cervix four-field box (soft tissue)—dose distribution	79	96	—	—
Cervix VMAT	99	100	—	—
Postmastectomy breast	44	91	—	—
Whole brain (MLC shielding)—field shape	76	100	87	100
Whole brain (MLC shielding)—dose distribution	67	99	73	96
Whole brain (rotated collimator)—field shape	73	93	—	—
Whole brain (rotated collimator)—dose distribution	51	79	—	—

Abbreviations: MLC, multileaf collimator; VMAT, volumetric-modulated arc therapy.

^a^ Approaches 1 and 2 represent different algorithms that serve as cross-checks on each other.

### Acceptability Testing and Statistical Plan

Previous studies have shown that there can be significant variation in what is considered clinically acceptable between radiation oncologists^18-20^; this can be the result of differences in style, training, local clinical approach, clinical trial paradigms, and other factors. Thus, to demonstrate reasonable clinical utility of the RPA, it is necessary to collect reviews from clinical professionals from multiple institutions.

Results from earlier studies indicated that we could expect close to 90% of automatically generated plans to be clinically acceptable.^26,27^ When accounting for the potential added variability of physicians from multiple centers, our working hypothesis was that at least 80% of automatically generated treatment plans would be considered clinically acceptable.

The clinical acceptability of each automated task was assessed for 75 separate patient test cases (for each subsite) by a minimum of three reviewers, each from a different institution (which would mean 25 reviews per reviewer). Each reviewer was asked to score using a 5-point Likert scale: (1) unusable, (2) major edits, (3) minor edits that are required, (4) minor edits that are not required (ie, stylistic differences), and (5) use as-is. For simple plans that are easily edited, a score of at least three was defined as clinically acceptable. For VMAT plans, which are more difficult to edit, a score of at least four was defined as clinically acceptable. Of note, results were considered acceptable only if they were considered safe (even if minor edits were not made). This analysis was done with institutional review board approval.

With 75 patient cases, each scored by one reviewer, if 67 (89.3%) cases receive an acceptable rating, then the corresponding exact 95% CI for the rate of acceptable plans will be (80.1% to 95.3%).

## RESULTS

### Reviewers

A group of 31 radiation oncologists from 16 different institutions, six countries, and five continents participated. Each case was reviewed by at least three radiation oncologists, each from a different institution. The radiation oncologists only reviewed cases for disease sites they routinely treated. For usability and other RPA development, an additional 29 residents, physicists, dosimetrists, and radiation therapists reviewed cases. However, their scores are not included in the final analysis.

### Review Process

**FIG 2:**
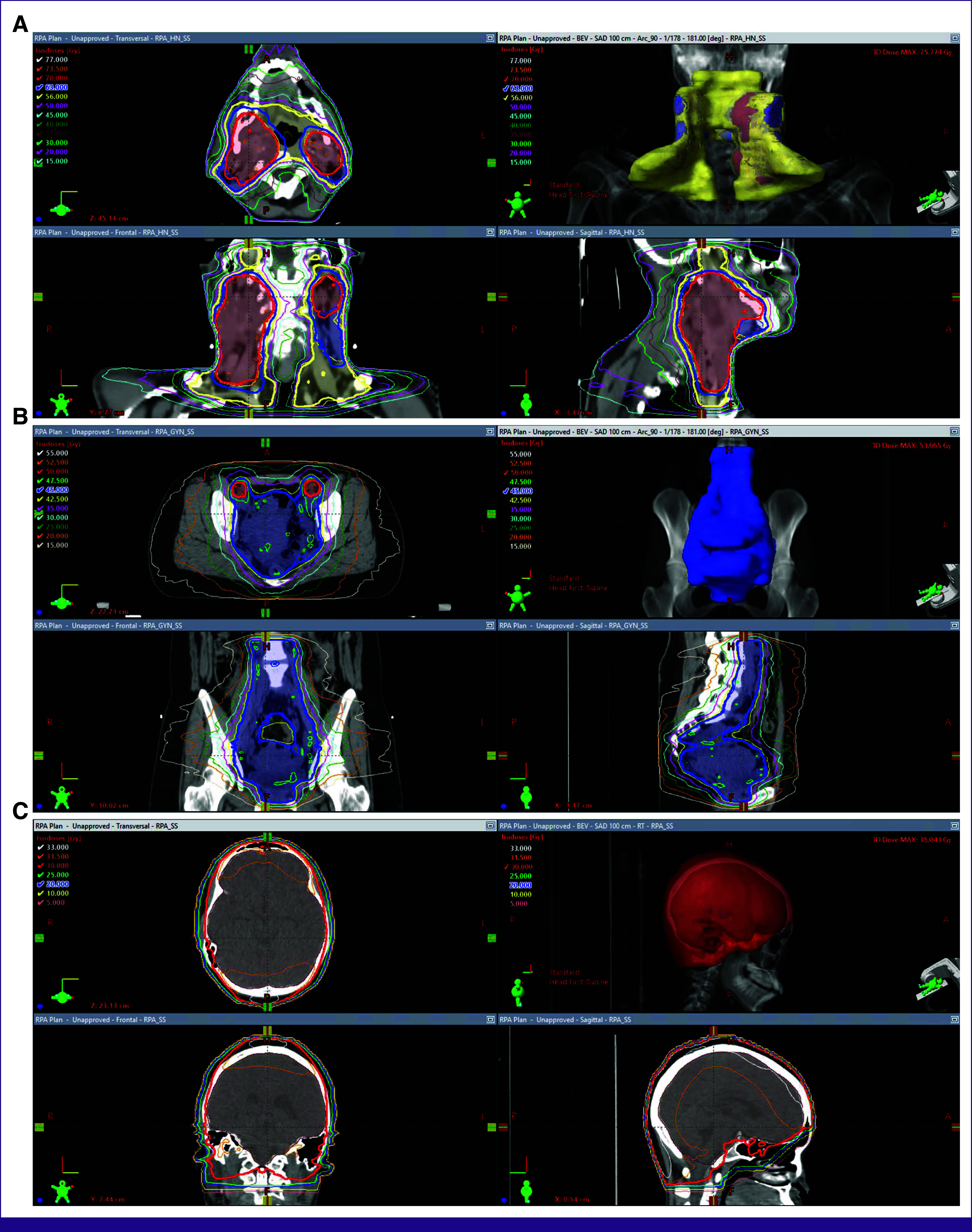
Example RPA-generated contours and plans: (A) head/neck, (B) cervix, and (C) whole brain.

Most reviews were performed in two to three 1-hour online sessions, hosted by a member of the RPA team, where the reviewer was given access to the anonymized patient data (examples shown in Fig 2). For simple cases (such as review of body contours or whole-brain apertures), reviewers also had the option to review plans on a pdf document.

### Review Results

**FIG 3:**
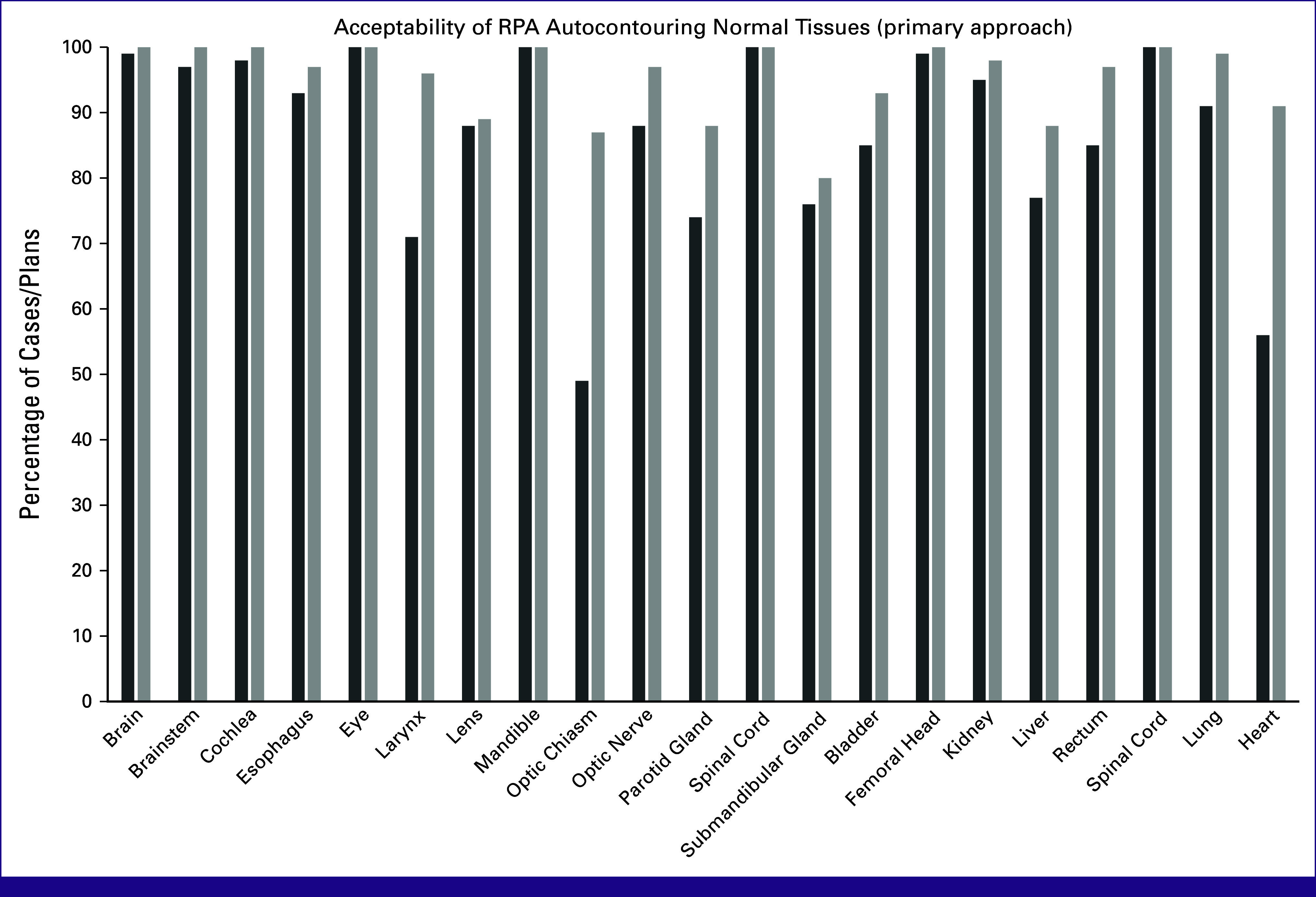
Acceptability of RPA autocontouring normal tissues. The percentage acceptable by physicians as-is (dark bars) and with minor edits (light bars) are shown for each normal tissue contoured with the primary approach.

**FIG 4:**
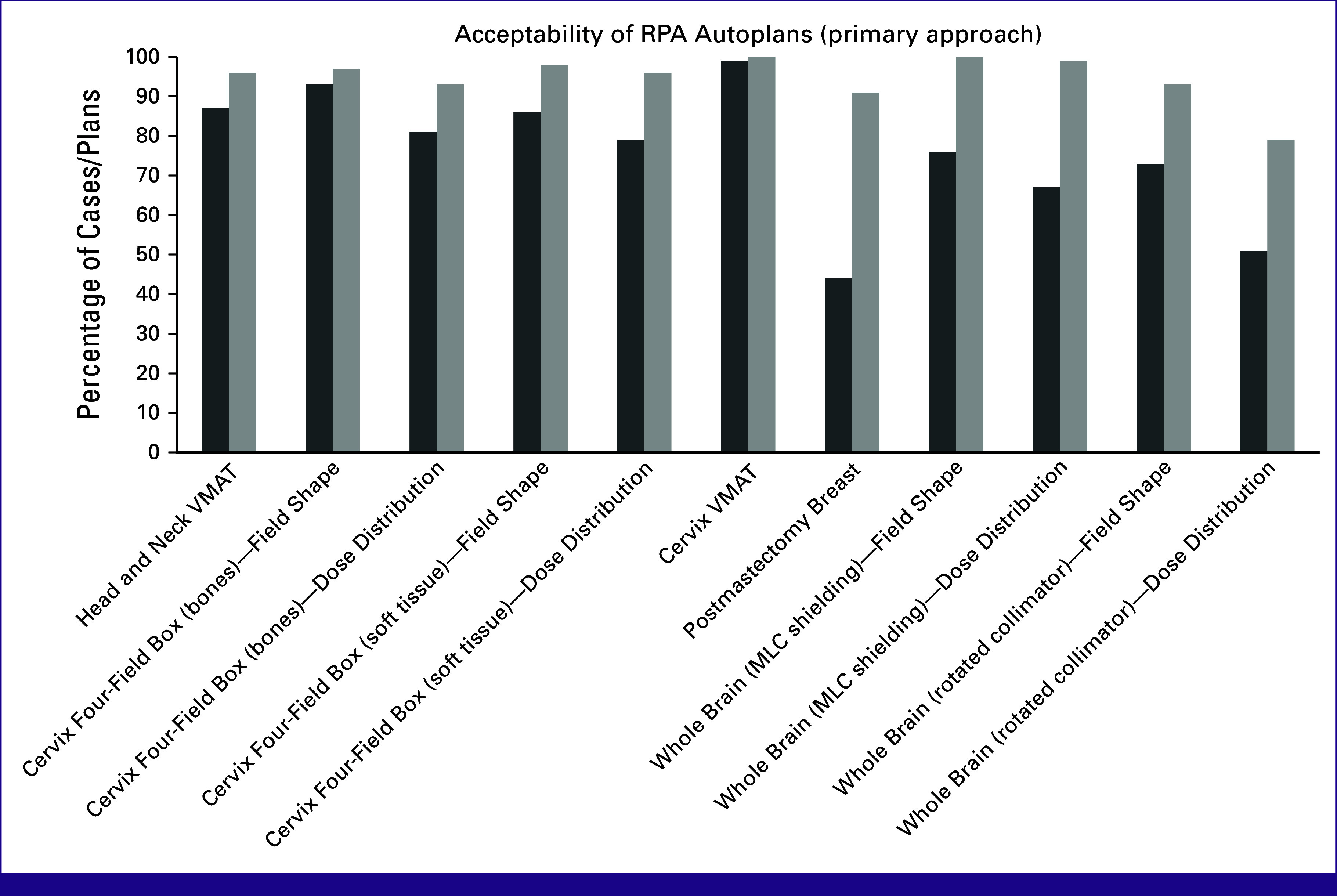
Acceptability of RPA autoplanning. The percent acceptable by physicians as-is (dark) and with minor edits (light) are shown for plans generated by the RPA with the primary approach. RPA, Radiation Planning Assistant; VMAT, volumetric-modulated arc therapy.

Acceptability results were generally high for normal tissues and targets (Table 1; Fig 3) as well as plans (Table 2; Fig 4). Some minor editing was recommended, especially for elective lymph node CTVs for head/neck planning. This is required by the RPA, as there is not yet sufficient ability for the RPA to contour GTV. Some editing of CTVs is, therefore, expected, especially in the vicinity of the GTV. This was also seen, but to a lesser degree, for the head/neck VMAT plans, where one reviewer scored 40% of plans as use as-is (96% use after minor edits), but the other reviewers scored 76%-96% of plans as acceptable as-is. This indicates a difference in the time-savings that are achievable, and how this will, to some extent, depend on how well aligned the planning philosophy of the radiation oncologist is with that of the RPA.

A similar observation was seen for the dose distribution for one of the whole-brain approaches, where one reviewer scored only 56% of plans as acceptable as-is or after minor edits, compared with 88% and 92% for the other two reviewers (79% overall). This indicates that the RPA-generated whole-brain approach is consistent with the clinical practice of two of the reviewers, and less so for the third (which could affect their use of this module of the RPA).

Acceptability did vary by technique. Acceptability results were generally high for VMAT plans. The results for conformal plans were presented in terms of the field shapes, which define the treatment volume, and the dose distribution. Overall results were good, but the dose distribution acceptability results were slightly lower; this is likely because the version of the RPA tested did not include the use of subfields to improve dose homogeneity. In other work, we have shown that automatically adding subfields results in higher levels of acceptability.^26,28,29^ Chest wall acceptability results were also lower for use as-is, although high after minor edits. This is consistent with our earlier work, where we showed that these plans are easily corrected to give clinically acceptable plans.

### Outliers

The reviewers were not asked to follow specific guidelines but to follow their own clinical practice. Autocontouring of the lymph node CTVs for head/neck treatments demonstrated noticeable interuser disagreement in the assessment of the clinical acceptability of the output of the RPA. For the lymph node CTV autocontouring, three of four radiation oncologists scored the autocontours as use as-is or after minor edits for 72%-100% of cases (depending on the specific lymph node level and the oncologist). However, one radiation oncologist scored lymph node CTVs as use as-is or after minor edits for only 12% of cases. These results indicate that there can be significant variations in what individual users will consider to be clinically acceptable. Most users accept the RPA contouring style (determined by the original training data) and approve the autocontours, but there will be some users who do not accept this clinical approach.

## DISCUSSION

The multiple contouring and treatment planning tasks in radiation oncology are uniquely suited for automation; the RPA was created to use AI approaches to improve efficiency and availability of high-quality RT in low-resource settings. However, metrics on contouring improvement and plan quality do not capture real-world scenarios; in the end, will physicians accept automated plans created by the RPA?

In this study, extensive physician reviews of RPA autocontours and plans demonstrate that these are largely acceptable as-is or with minor edits (considered style differences without impact on safety or disease control). This is extremely important, showing that the output of the RPA is not only safe but clinically relevant to practitioners for patient care.

Automated approaches to RT tasks are increasingly being developed. The success of these is typically demonstrated by similarity metrics, such as Dice similarity coefficient (DSC) and Hausdorff distance, and dose-volume histogram metrics. These metrics do not necessarily mirror physician acceptability.^30^ Understanding the best practices for creation of a data set and evaluation has been the subject of extensive testing in recent years.

One key component to automated approaches is training cases on high-quality data and extensive testing of the AI-generated results. Tryggestad et al^31^ demonstrated that data curation is important; a team devoted over 6,000 person-hours to carefully curate a series of 490 patient cases. Amjad et al created a curated set of 42 organs at risk for head/neck, abdomen, and male pelvis contours for five autosegmentation models. These had a high rate of DSC and physician acceptability,^32^ and automation reduced contouring time by 88% for the male pelvis, 80% for head/neck, and 65% for abdominal models. Byun et al^33^ used 11 experts from two institutions to serve as the basis for nine organs at risk in 10 cases of breast cancer (intact); the resultant 110 manual contours were compared with autocontours and physician-edited autocontours, demonstrating a high DSC, acceptability, and reduced time to final contours through the use of automation. Duan et al^34^ evaluated prostate cancer autocontours and autoplans, showing 95.7% of autocontours were scored as perfect (34.8%) or acceptable (60.9%). Interestingly, 39.6% of the autocontours were considered equal or better than the reference contours.

Our work is consistent with these previous experiences, demonstrating the ability of well-trained AI approaches to create normal tissue autocontours and robust RT plans. However, these previous analyses do not provide end-to-end planning services (such as the one-step creation of cervical cancer plans using four-field box and soft tissue contouring or whole brain) and elective target coverage contouring and planning (such as the two-step creation of cervical cancer VMAT and head/neck VMAT plans). In this analysis, the RPA is shown to provide high-quality end-to-end autoplanning and contouring results that are clinically acceptable to physicians.

These data do show some potential concerns regarding AI-based contouring and planning. The scoring demonstrated that interobserver variability and physician style affects opinions on clinical contouring and planning. For instance, one physician did not find the head/neck contours and plans to be acceptable. The acceptability by the remainder of the physicians does suggest that this individual may be an outlier, but it still highlights the known physician variability.^18-20^ Once the RPA is deployed clinically, we will gain experience on how much physician variability affects clinical use and develop strategies to mitigate this. Another potential issue to be explored will be practicality and user-friendliness of the RPA. In terms of overall interest, previous work from our group demonstrated overwhelming enthusiasm in the RPA (86.7%) and anticipation of usability within 2 years (80%)^35^; this survey also showed 83.4% believed it would improve their clinical workflow. We look forward to clinical implementation in 2023-2024 to understand more practical aspects to ensure usability and uptake.

Despite the high degree of clinical acceptability in this work, there are still potential limitations to clinical deployment for actual patients. In the study by McIntosh et al, researchers extensively tested automated planning for prostate cancer with clinical acceptability of the machine learning plans in 92% of cases in the testing phase; however, on clinical deployment, the selection of the machine learning plans dropped from 83% to 61%.^36^ This highlights that further testing is needed, and ultimately, physicians will choose what they feel is the best plan for their patient, regardless of its source.

In summary, this work demonstrates that the RPA can generate automated contours for CTVs and organs at risk, treatment fields, and plans for a wide variety of cancer types that are clinically acceptable as-is or with minor edits in the majority of cases. With recent FDA 510(k) clearance, future work will be to transition this to clinical practice in centers around the world to establish true clinical implementation and practicality. These data will be crucial to adapt the system, with regard to contouring/planning and workflow/usability, to improve RT to low-resource settings around the globe.

## APPENDIX. Radiation Planning Assistant Consortium

Shareen Ahmad, David Anderson, Arjig Baghwala, Karen Chan, Prajnan Das, Albert Edwards, May Elbanna, Hesham Elhalawani, Medhat Elsayed, Agnes Ewongwo, Nazia Fakie, C. David Fuller, Adam Garden, Matt Gove, Teresa Guerrero-Urbano, Njeri Kaittany, Mishal Khan, Joshua Langer, Percy Leeig, Becky Lee, Anna Lee, Belinda Lee, Michelle Leech, Ben Li, Katie Lichter, Lilie Lin, Stacy Lin, Dorothy Lombe, Indranil Mallick, Sean Maroongroge, Rachael Martin, Gwendolyn McGinnis, Megan Mezera, Mustefa Mohammedsaid, Son Nguyen, Jenny Nuanjing, Tony Phillips, Surendra Prajapati, Lydia Punt, Valerie Reed, Dominique Roniger, Simona Shaitelman, Alicia Sherriff, Jay Shiao, Heath Skinner, Ashely Susan, Jordan Sutton, Hamza Syed, Sandy Thang, Juanita Thompson, Gary Walker, Julie Wetter, Ingrid White, Melody Xu, Yousif Yousif, Simeng Zhu
