# Erratum: Artificial Intelligence–Based Radiotherapy Contouring and Planning to Improve Global Access to Cancer Care

**DOI:** 10.1200/GO.24.00124

**Published:** 2024-04-04

The article by Court et al entitled “Artificial Intelligence–Based Radiotherapy Contouring and Planning to Improve Global Access to Cancer Care” (*JCO Global Oncology* 10.1200/GO.23.00376) was published online March 14, 2024, with an error.

The consortium list provided as an Appendix included two typos: Arjig Baghwala should be “**Arjit Baghwala**” and Percy Leeig should be “**Percy Lee.**”

This has been corrected as of April 4, 2024. The authors apologize for the error.

